# Supplementary material for: Optical Writing and Electro-Optic Imaging of Reversible Space Charges in Semi-Insulating CdTe Diodes
Source: Sensors (Basel). 2022 Feb 17;22(4):1579. doi: 10.3390/s22041579 (PMC8878099; doi:10.3390/s22041579)
Supplement: Supplementary file 1 [file sensors-22-01579-s001.zip › sensors-1592589-supplementary/sensors-1592589-supplementary.pdf]

Supplementary Materials

Optical Writing and Electro-Optic Imaging of Reversible Space Charges in Semi-insulating CdTe Diodes

Adriano Cola[1*], **Lorenzo Dominici**[2], **Antonio Valletta**[3]

[1] Institute for Microelectronics and Microsystems, IMM-CNR, Via Monteroni, 73100, Lecce, Italy
[2] Institute of Nanotechnology, NANOTEC-CNR, Via Monteroni, 73100, Lecce, Italy
[3] Institute for Microelectronics and Microsystems, IMM-CNR, Via Del Fosso Del Cavaliere, 100, 00133, Rome, Italy
* corresponding: adriano.cola@cnr.it

1. Carrier generation rate in CdTe

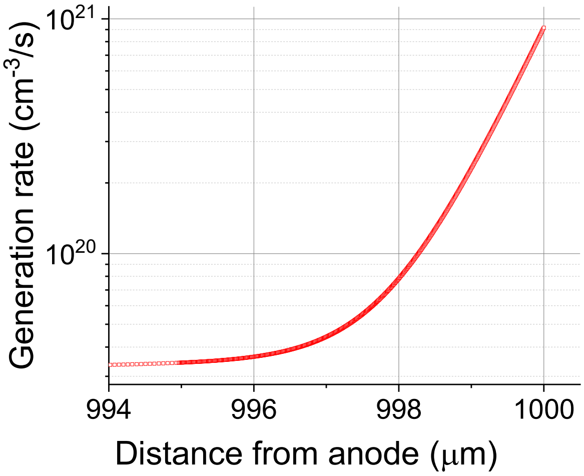

Figure S1. Typical profile of the carrier generation rate based on the specific characteristics of the incident light and on the optical absorption in CdTe (light is coming from the right).

2. Optical irradiations at different Temperatures

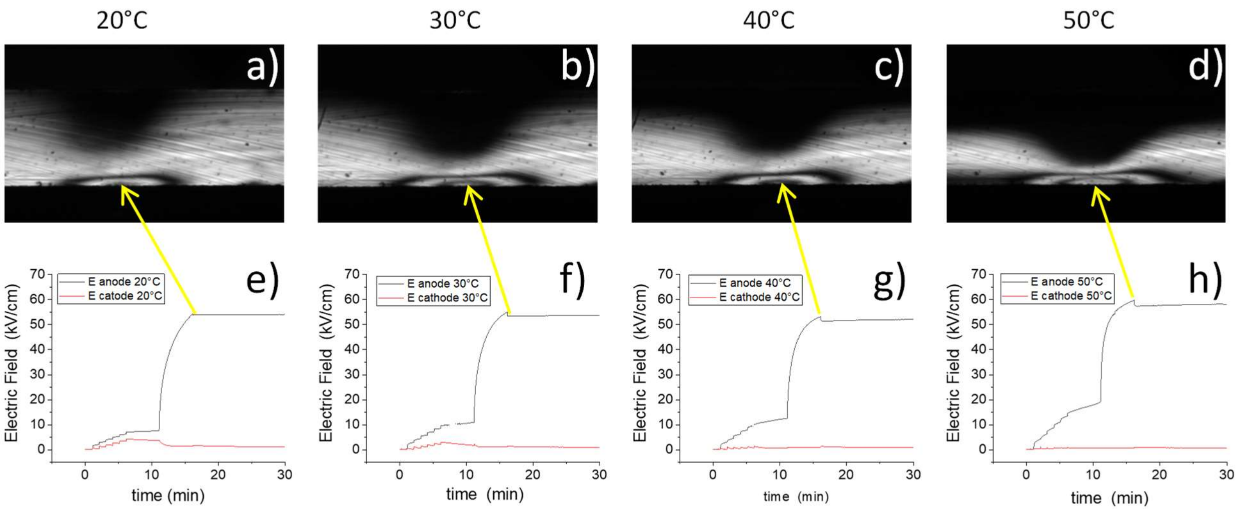

Figure S2 Measurements at different Temperatures: Pockels images just after the optical irradiation (upper panels) and electric field evolutions at the anode and cathode (lower panels). Yellow arrows highlight the spatial and temporal correspondence. Panels (**a**),(**e**) are at 20°C, (**b**),(**f**) at 30°C, (**c**),(g) at 40°C, and (**d**),(**h**) at 50°C. Neutral filters of optical density 2 × 10^{-2} have been used.

3. Two optical irradiation experiment

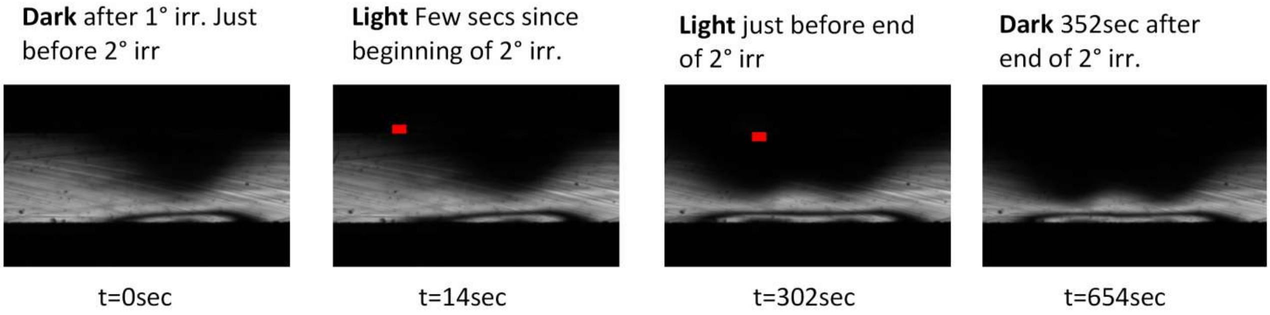

Figure S3. Pockels images acquired along the sequence of a two optical irradiation experiment. Relative times and conditions are labelled. (**a**) In the dark just before the 2° irradiation, (**b**) under light, few seconds since the beginning of second irradiation; (**c**) under light just before switching off the 2° irradiation; (**d**) under dark, few minutes after the end of the second irradiation. Red box in (**c**) and (**d**) refer to the incident light position on the cathode side for the second optical irradiation.

4. Simulated electric field profiles (anode to cathode)

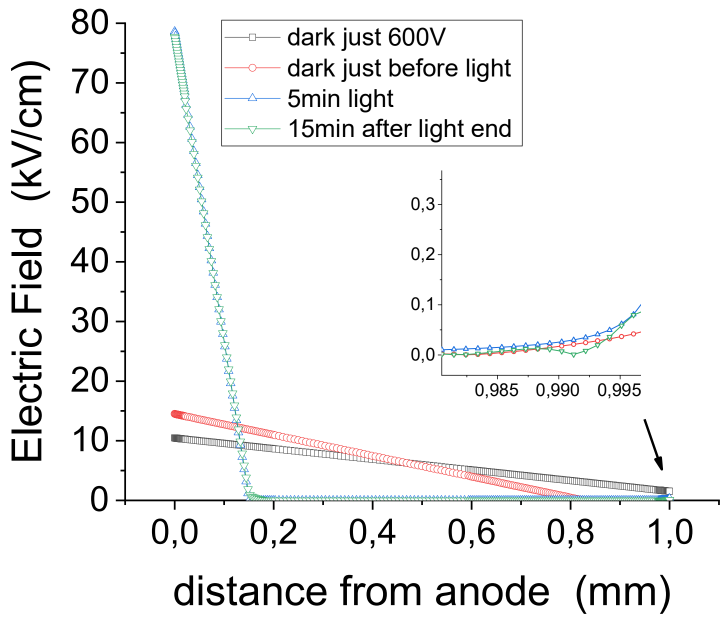

Figure S4. Simulation results corresponding to the vertical electric field profiles at different instants reported in Figure 3. In the inset, a zoom of data close to the cathode.

5. Maps of the x-y electric field components

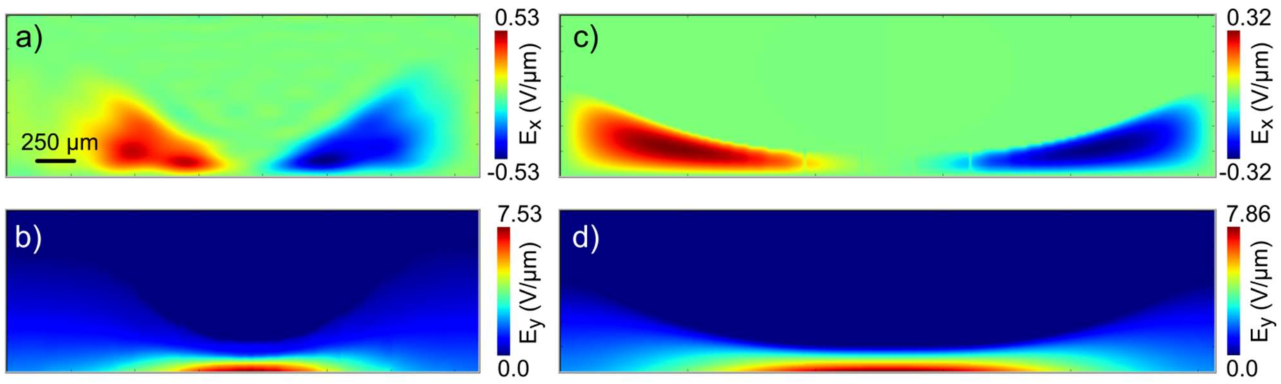

Figure S5. The electric field components E_x, E_y (**a**,**b**) from the experiments shown in Figure 5 and (**c**,**d**) from the numerical simulations in Figure 7. In the experimental case the E_y component is directly extracted from the fit of electro-optic measurements while the E_x is deduced from the E_y components by using the conservative feature of the electric field.

6. Simulated charge concentration and current density profiles

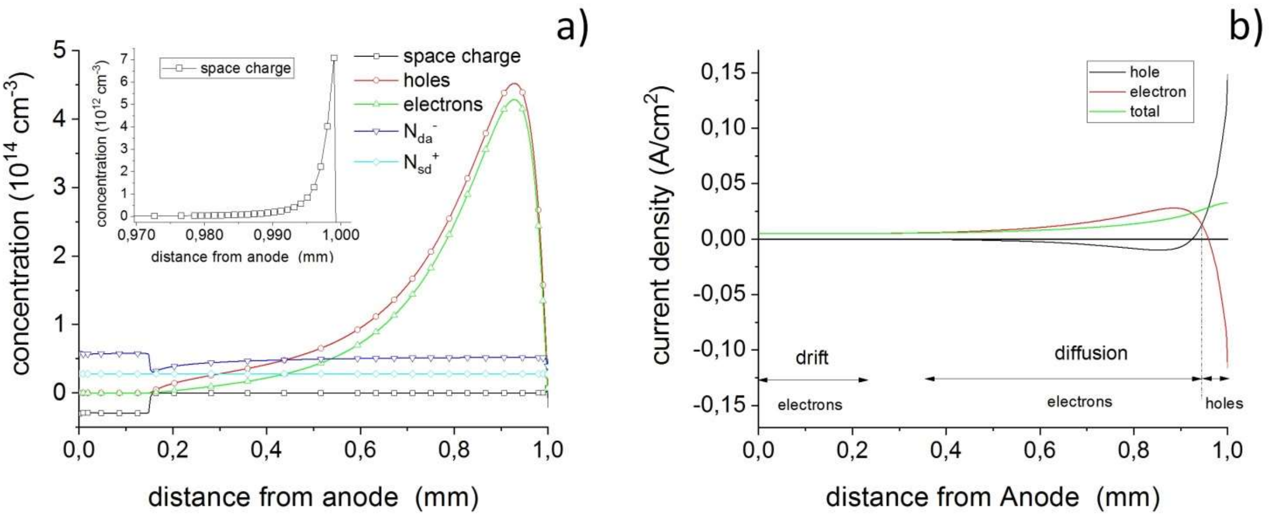

Figure S6. Simulated vertical profiles at the end of the optical irradiation, same parameters as in Figure 7. (**a**) Concentrations of holes, electrons, negatively ionized deep acceptor and positively ionized shallow donor. Their sum, i.e. the space charge concentration, is also reported. In the inset, a zoom of the space charge data close to the cathode. (**b**) Electron, hole and total current densities. Starting from cathode, the arrows denote the dominance the of hole diffusion, electron diffusion and, near the anode, electron drift.
