# Supplementary material for: Optical Writing and Electro-Optic Imaging of Reversible Space Charges in Semi-Insulating CdTe Diodes
Source: Sensors (Basel). 2022 Feb 17;22(4):1579. doi: 10.3390/s22041579 (PMC8878099; doi:10.3390/s22041579)
Supplement: Supplementary file 1 [file sensors-22-01579-s001.zip › sensors-1592589-supplementary/Video Abstract.pdf]

Video Abstract

The video shows the sequence of a typical irradiation experiment, the same as in Figs. 3 and 5 (T = 40°C, optical density 0.2). The initial frames show the transmission image between parallel polarizers (P_{para}), shown as a reference and used to normalize the successive Pockels images.
The Pockels images are acquired every 2 secs, starting with a zero bias, then the detector, still under dark, is biased in steps of 100V up to 600V. After that, the detector is irradiated for 5 mins from the cathode side by a line-focused optical beam (transverse size Δx = 150μm, see the red arrow for its approximate position). After irradiation, the light is switched-off and the device kept at 600 V for further 30 mins, then a backward voltage sweep returns to a 0 bias. The fringes appearing during the optical irradiation are due to the high electric field build up close to the anode, below the irradiated region. The video clearly shows that the perturbation of the electric field remains written all along the 30 mins after the optical irradiation. Such perturbation is erased when nulling the applied voltage, refreshing the device for a successive experiment with the same results.
